# Necroptosis Identifies Novel Molecular Phenotypes and Influences Tumor Immune Microenvironment of Lung Adenocarcinoma

**DOI:** 10.3389/fimmu.2022.934494

**Published:** 2022-07-14

**Authors:** Chen Zhao, Kewei Xiong, Abdalla Adam, Zhiqiang Ji, Xiangpan Li

**Affiliations:** ^1^ Department of Oncology, Renmin Hospital of Wuhan University, Wuhan, China; ^2^ School of Mathematics and Statistics, Central China Normal University, Wuhan, China; ^3^ School of Medicine, Wuhan University, Wuhan, China; ^4^ School of Public Health, Cheeloo College of Medicine, Shandong University, Jinan, China

**Keywords:** lung adenocarcinoma, necroptosis, tumor immune microenvironment, immunotherapy, multi-omics

## Abstract

This study aims to investigate the immune and epigenetic mutational landscape of necroptosis in lung adenocarcinoma (LUAD), identify novel molecular phenotypes, and develop a prognostic scoring system based on necroptosis regulatory molecules for a better understanding of the tumor immune microenvironment (TIME) in LUAD. Based on the Cancer Genome Atlas and Gene Expression Omnibus database, a total of 29 overlapped necroptosis-related genes were enrolled to classify patients into different necroptosis phenotypes using unsupervised consensus clustering. We systematically correlated the phenotypes with clinical features, immunocyte infiltrating levels, and epigenetic mutation characteristics. A novel scoring system was then constructed, termed NecroScore, to quantify necroptosis of LUAD by principal component analysis. Three distinct necroptosis phenotypes were confirmed. Two clusters with high expression of necroptosis-related regulators were “hot tumors”, while another phenotype with low expression was a “cold tumor”. Molecular characteristics, including mutational frequency and types, copy number variation, and regulon activity differed significantly among the subtypes. The NecroScore, as an independent prognostic factor (HR=1.086, 95%CI=1.040-1.133, p<0.001), was able to predict the survival outcomes and show that patients with higher scores experienced a poorer prognosis. It could also evaluate the responses to immunotherapy and chemotherapeutic efficiency.

In conclusion, necroptosis-related molecules are correlated with genome diversity in pan-cancer, playing a significant role in forming the TIME of LUAD. Necroptosis phenotypes can distinguish different TIME and molecular features, and the NecroScore is a promising biomarker for predicting prognosis, as well as immuno- and chemotherapeutic benefits in LUAD.

## Introduction

Lung cancer is the leading cause of cancer-associated death worldwide due to its high recurrence rate and inopportune diagnosis (1). The majority of these patients are non-small cell lung cancer, categorized into several histological types (2). Among them, lung adenocarcinoma (LUAD) is the prevalent subtype, accounting for nearly 60% of the cases, and the rate is still increasing (3, 4). Although patients with early-stage cancer demonstrate higher survival rates in general, the percentage of deaths can reach up to 44% within 5 years after surgical resection (5). With the development of next-generation sequencing technology, targeted therapy for LUAD has been widely applied over the last decade, especially targeting ALK, EGFR, KRAS, and RET (6–8). For advanced LUAD, treatment is majorly based on anti-PD-1/PD-L1 mono-immunotherapy or combination strategies with chemotherapeutics (9). Despite these advances in molecular biology and personalized treatment, the 5-year overall survival (OS) rate of patients with LUAD remains at 15% (10). Therefore, screening for specific diagnostic and prognostic biomarkers for LUAD patients is essential to discover novel therapeutic targets and enhance patients’ survival time.

Necroptosis is a programmed cell death mediated by RIP1 kinase and RIP3, characterized by massive leakage of cytosolic contents and organelle swelling. Increasing evidence has shown that necroptosis plays an indispensable role in cancer pathogenesis (11). Transcriptional changes of necroptosis regulatory factors greatly influence the prognosis of cancers such as breast cancer, colorectal cancer, chronic lymphocytic leukemia and glioblastoma, reportedly (12–15). Compared with apoptotic cells, necroptotic cells are remarkably more immunogenic due to their robust inflammatory responses (16). Due to the defect in the necroptosis components, cancer patients exhibit weak immunogenic neoplastic cells and develop strong immune evasion capacities (17). Seifert el al. found that the immune-suppressive tumor microenvironment (TME) correlating with the RIP1/RIP3 signaling pathway depended partly on the necroptosis-induced chemokine attractant CXCL1 expression level, the blockade of which protected against pancreatic ductal adenocarcinoma (18).

Conversely, the induction of necroptosis combined with immune checkpoint inhibitors (ICIs) can enhance anti-tumor activity, even in ICI-resistant tumors (19). Several research lines have verified that necroptosis can recruit inflammatory immune cells dedicated to promoting the tumor in neoplasia (20). Namely, necroptosis has dual roles in cancer biology. Firstly, by increasing dendritic cells, it can activate cytotoxic T cells to suppress tumor proliferation. Secondly, it can promote angiogenesis and genomic instability through cytokines and reactive oxygen species to cause tumor progression (21).

Although necroptosis has complex roles, the aforementioned studies only included a few (especially one or two) necroptosis-related molecules, while there are a variety of interconnected factors that can trigger the interaction between necroptosis regulatory systems and TME immune responses. To our knowledge, only a few studies have investigated necroptosis-related molecules in tumors, especially in gastric cancer. Wang et al. constructed a necroptosis-related mRNA-based signature and a lncRNA-miRNA-mRNA regulatory axis (22). Zhao et al. developed a prognostic model based on 16 necroptosis-related lncRNAs and identified molecular phenotypes to distinguish the cold and hot tumors in gastric cancer (23). However, the thesis did not show the multi-omic features of necroptosis-related genes and lacked model validation with an independent cohort. Moreover, the measurement of immune differences between the phenotypes was insufficient because it only engaged with immunocyte infiltrating levels and expressions of immune checkpoints. Hence, it is crucial to comprehensively analyze the necroptosis patterns in multi-omic aspects and explore the association between different necroptosis regulatory molecules and TME.

This study involved 30 necroptosis-related genes to show their heterogeneity in pan-cancer, including changes in transcriptional expression, somatic mutation, copy number variation, and DNA promoter methylation. We identified three molecular phenotypes using unsupervised clustering to correlate tumor necroptosis with TME and molecular variations in LUAD. A reliable prognostic scoring system named NecroScore was constructed to quantify the necroptosis molecules in LUAD and propose potential therapeutic strategies for different stratifications, enhancing prognosis and implementing more personalized precision medicine in clinical practice.

## Materials and Methods

### Data Sources and Preprocessing

The workflow of the study is shown in **Supplementary Figure 1**. A total of 1,064 patients were enrolled for analysis, and the specific description of data sources was provided in the **Supplementary Method**. All the data used in this study is freely accessible to the public, mainly derived from The Cancer Genome Atlas (TCGA, https://portal.gdc.cancer.gov/) and the Gene Expression Omnibus (GEO, https://www.ncbi.nlm.nih.gov/geo/), the UCSC Xena (https://xenabrowser.net/datapages/) and the cBioPortal (https://www.cbioportal.org/). Transcriptional profiles of fragments per kilobase per million (FPKM) were transformed into transcripts per kilobase per million (TPM, and batch effects were corrected using the ComBat method. More details are provided in the **Supplementary Method**. The present study honored the data access policies of each database.

### The Construction of Necroptosis Regulator Phenotypes

Based on the expression levels of necroptosis-related molecules, unsupervised average linkage K-means clustering analysis was utilized to identify novel necroptosis phenotypes in LUAD. The optimal clusters were determined by the cumulative distribution function (CDF), which declined the slowest. We then depicted the immune microenvironment and genomic characteristics among the molecular phenotypes. More details are documented in the **Supplementary Method**.

### Development of a NecroScore Prognostic System

We adopted the PCA algorithm to create a scoring system based on differentially expressed necroptosis regulators among the clusters in LUAD named NecroScore according to the formula:


NecroScore=∑(PC1+PC2)

where *PC*1 represents the largest proportion of the variance in the initial expression lineage to be decomposed, followed by *PC*2. We examined the distribution differences in LUAD phenotypes and explored the clinical, tumor immune microenvironment, and mutation characteristics to validate the effectiveness of the NecroScore. More details are documented in the **Supplementary Method**.

### Statistical Analysis

All statistical and bioinformatics analysis was performed in R (https://www.r-project.org/). Mann-Whitney U test (also known as Wilcoxon rank-sum test) for non-normally distributed continuous data and unpaired t-test for normal distributed continuous data were used to estimate the difference between the two groups. To compare three or more groups, Kruskal-Wallis (KW), one-way ANOVA, and Welch one-way ANOVA were applied. As to the categorical data, Fisher’s exact test was employed in the present study. Detailed methods are stated in the **Supplementary Method**.

## Results

### Epigenetic and Transcriptional Characteristics of Necroptosis Regulators

**Figure 1:**
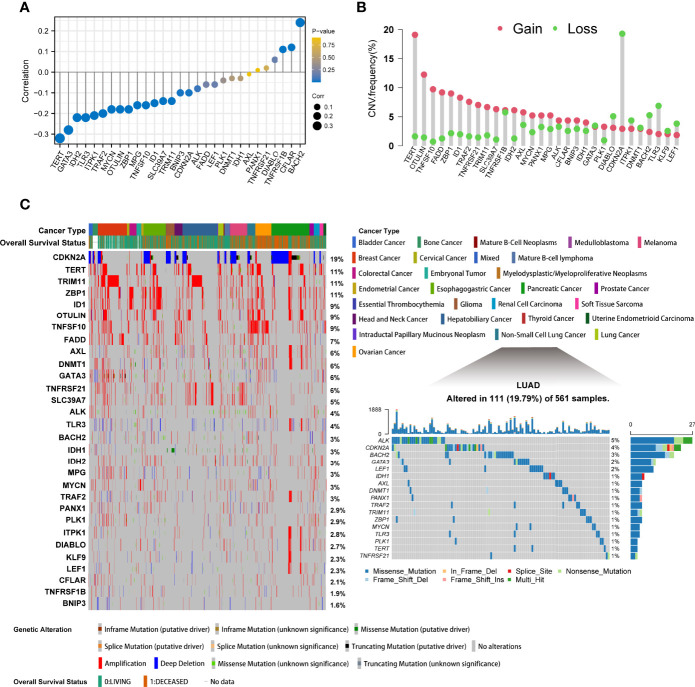
The epigenetic alteration landscape of necroptosis molecules in lung adenocarcinoma. **(A)** The lollipop chart showed the correlations between DNA promoter methylation and mRNA expression levels. The circle size represented the correlation coefficients. Bluer dots indicated a lower p-value. **(B)** The CNV frequency of necroptosis regulators in TCGA cohort. The height of bars represented the alteration frequency. Pink represented CNV amplifications (gain), and green dot represented CNV deletions (loss). **(C)** The left part showed the mutant frequency and types in 28 cancers from cBioPortal. Each column indicated individual patients. The upper band showed cancer types and vital status. The number on the right displayed the mutation frequency of each gene. The right part illustrated the somatic genetic mutation landscape in lung adenocarcinoma in TCGA. The upper barplot showed the overall mutation frequency of each sample. The right stacked barplot demonstrated the proportion of each variant type. Only molecules having mutations, namely mutant frequency > 0%, were illustrated in the waterfall plot.

A total of 30 differentially expressed necroptosis-related genes were identified in the TCGA-LUAD cohort by the R package “DESeq2” (**Supplementary Table 1**). The 30 molecules were ALK, AXL, BACH2, BNIP3, CDKN2A, CFLAR, DIABLO, DNMT1, FADD, GATA3, ID1, IDH1, IDH2, ITPK1, KLF9, LEF1, MPG, MYCN, OTULIN, PANX1, PLK1, SLC39A7, TERT, TLR3, TNFRSF1B, TNFRSF21, TNFSF10, TRAF2, TRIM11, and ZBP1. The top-left part of **Supplementary Figure 1** shows a link of the regulatory process in necroptosis. To reveal the epigenomic features of necroptosis regulators in LUAD, we analyzed methylation levels. It was demonstrated that significant negative correlations were observed between DNA promoter methylation and transcriptional expression levels of TERT and GATA3, while BACH2 had weak positive associations with its methylation (**Figure 1A**). Since CNV can influence the whole genome at arm-level or alter a broad region at a focal-level, sparking larger influences on somatic mutations, we further depicted the CNV landscape of these genes. The exploration of copy number values demonstrated aberrant alterations in 30 regulators, where TERT and OTULIN had a widespread frequency of CNV amplification, while CDKN2A exhibited a significant CNV deletion in LUAD (**Figure 1B**). In pan-cancer mutation analysis according to cBioPortal, CDKN2A showed the highest mutation types with deep deletions, followed by TERT, TRIM, and ZBP with a frequency of 11% amplifications. Of the 561 samples of LUAD, 111 experienced mutations of necroptosis-related genes with a frequency of 19.79%. However, it was illustrated that ALK had the highest mutation frequency, followed by CDKN2A, and splice sites were only observed in CDKN2A and IDH1 (**Figure 1C**).

**Figure 2:**
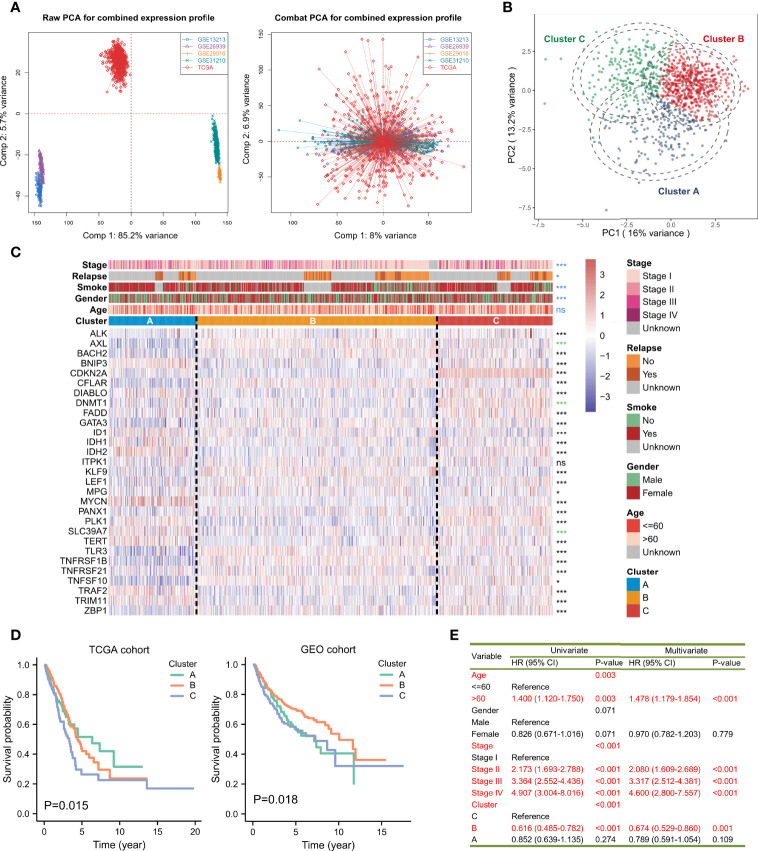
Average-linked and K-means-based unsupervised clustering to determine three necroptosis phenotypes. **(A)** Principal component analysis (PCA) showed the distribution of transcriptional expressions of the 29 necroptosis-related genes in five lung adenocarcinoma cohorts before (left part) and after (right part) the batch effect correction. **(B)** PCA indicated that the expression profiles of the 29 necroptosis molecules could distinguish necroptosis phenotypes in the TCGA and GEO-meta cohorts. **(C)** The heatmap showed the correlations between the three necroptosis phenotypes and clinicopathologic features, and the expression changes of the 29 necroptosis-related genes. Asterisks represented p-value (***p < 0.001, *p < 0.05 and ns was the abbreviation of no significance), the color of which represented methods of statistical tests. Blue denoted Fisher’s exact test, black denoted Kruskal-Wallis test, and green denoted Welch one-way ANOVA test. The inner color of each cell demonstrated the expression variations. Redder was connected with overexpressed levels and bluer was linked with lower levels. **(D)** Kaplan-Meier curves and log-rank test showed significant differences of survival rate differences among the three necroptosis clustering subtypes in the TCGA and GEO-meta cohorts, respectively. **(E)** A univariate and multivariate Cox model revealed the prognostic influences of necroptosis subtypes on overall survival outcomes in the TCGA cohort.

It was found that most of the regulators exerted significant expression differences between the para-cancerous and tumor tissues of LUAD. Several genes were found at lower expression levels in tumor cases, such as AXL, BAHC2, CFLAR, and DNMT1 while others were overexpressed such as BNIP3, DIABLO, FADD, and IDH1 (**Supplementary Figure 2A**). In addition, BNIP3, DIABLO, DNMT1, FADD, LEF1, PANX1, PLK1, TNFRSF21, and ZBP had significantly changed expressions in different pathologic stages from I to IV (**Supplementary Figure 2B**). Hierarchical clustering was performed for the 30 genes and four clusters were obtained: Cluster A (TERT, MYCN and ALK), Cluster B (AXL, BNIP3, CFLAR, DIABLO, DNMT1, FADD, ID1, ITPK1, KLF9, MPG, PANX1, TLR3, TNFRSF1B, TRAF2, TRIM11), and Cluster D (BACH2, CDKN2A, GATA3, LEF1, PLK1 and ZBP1). To fully understand the regulatory mechanism of necroptosis-related genes in LUAD, we collected 1,064 samples and corrected the batch effect using the ‘ComBat’ method. The baseline of the five cohorts, before and after the correction, is shown (**Figure 2A**). It can be seen that the sum of components 1 and 2 decreased from 90.9% to 14.9%. The conglomerate distributions also indicate that the batch effect was roughly removed. A comprehensive landscape of necroptosis molecules interplaying with genetic similarity and their prognostic values for LUAD patients is depicted with a network diagram (**Supplementary Figure 2C**). We found that the necroptosis regulators in the same category present remarkably positive associations in transcriptional expression levels, and significant correlations were demonstrated among the four modification patterns. According to the log-rank test, over-expressed DIABLO, FADD, ID1, MPG, TRIM11, PLK1, TNFRSF21, and TERT were associated with poor overall survival outcomes, namely risk factors, while ALK was considered as a favorable factor. A univariate Cox model showed that FADD, PANX1, PLK1, TRIM11, TRAF2, TERT, and ID1 might serve as risk factors, while ALK showed a preventative effect on the prognosis (**Supplementary Figure 2D**). The abovementioned results strongly imply that the imbalance across the necroptosis regulatory genes is correlated with the tumorigenesis of LUAD.

### Novel Subtypes of LUAD Identified by Unsupervised Clustering

The K-means-based unsupervised learning method was applied to classify patients with 29 (OTULIN was not detected in the combined expression matrix after the batch effect correction and since excluded) necroptosis regulatory molecules. According to the distribution of different clusters and the cumulative distribution function (CDF), the optimal clusters and three distinct necroptosis phenotypes were eventually determined, including 210 cases in Cluster A, 578 cases in Cluster B, and 276 cases in Cluster C (**Supplementary Figure 3A-C** **;** **Supplementary Table 2**). PCA confirmed that the three clustering subtypes could be distinguished by the 29 necroptosis-related molecules in expression levels (**Figure 2B**). A heatmap visualized the expression changes of necroptosis regulators. Necroptosis-related genes exhibited the highest expression levels in Cluster C, followed by Cluster B, and then Cluster A (**Figure 2C**). The distribution of clinicopathologic phenotypes, including age, gender, smoking history, relapse situation, and pathologic stage, was also examined by Fisher’s exact test in the three necroptosis clusters, simultaneously. We found that Clusters B and C had more women patients (p < 0.001). Patients in Cluster A were more likely to smoke (p < 0.001). Patients in the early stages (Stage I or II) were mostly from Cluster B (p < 0.001). Nevertheless, the age of patients presented no statistical significance in the distribution among the phenotypes. KM survival analysis for the three main necroptosis phenotypes revealed a relatively prominent prognostic advantage in Cluster B (**Figure 2D**). The univariate and multivariate Cox proportional hazard regression models indicated that Cluster B could predict overall survival in LUAD (Univariate: HR = 0.616, 95%CI = 0.485-0.782, p < 0.001; Multivariate: HR = 0.674, 95%CI = 0.529-0.860, p = 0.001; **Figure 2E**) along with Cluster C. Moreover, age and stage also served as underlying predictors for survival.

### TME Immune Variations in Distinct Necroptosis Phenotypes

**Figure 3:**
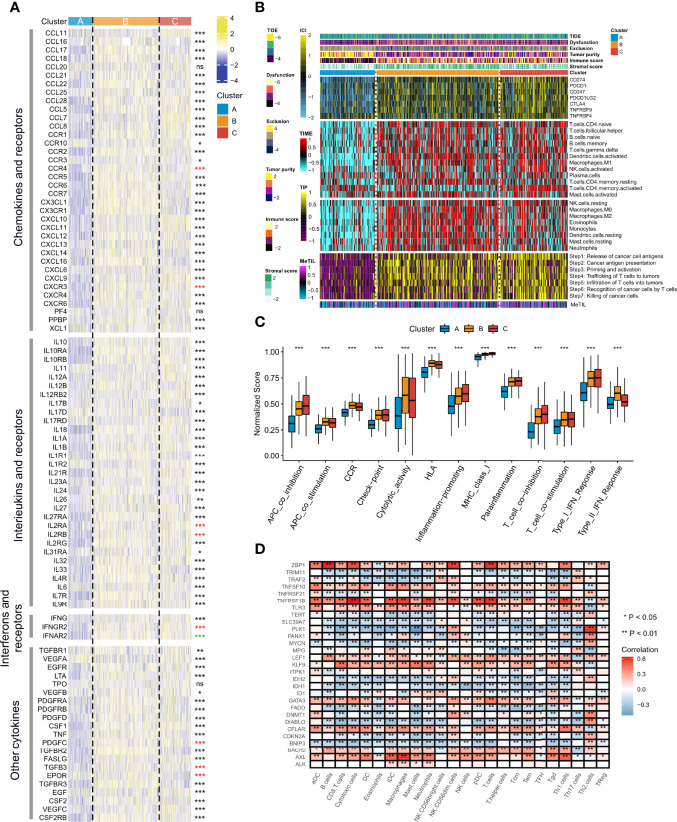
Alterations of immunomodulators and several quantified types of tumor immune microenvironment among the three necroptosis phenotypes. **(A)** The heatmap showed changes of mRNA expression levels of chemokines, interleukins, alterons and their corresponding receptors, and other cytokines among the three necroptosis subtypes. Asterisks represented p-value (***p < 0.001, **p < 0.01, *p < 0.05, and ns was the abbreviation of no significance), the color of which represented methods of statistical tests. Black denoted Kruskal-Wallis test, red denoted one-way ANOVA test, and green denoted Welch one-way ANOVA test. **(B)** The composite heatmap showed the tumor immune microenvironment profile in the TCGA-LUAD cohort, with the top panel showing the variations of several immune checkpoints, the middle panel showing enrichment scores of 24 immune cell types, and the relative bottom panel showing the enrichment levels of seven steps in the anti-tumor immune cycle. The DNA methylation level of tuWmor-infiltrating lymphocyte (MeTIL) was stuck in the lowest part. The TIDE score, dysfunction score, exclusion score, tumor purity, immune score, and stromal score were annotated at the top of the heatmap. **(C)** The variations of normalized scores of 13 immune pathways were revealed by Kruskal-Wallis test. **(D)** The heatmap showed the associations between the 29 necroptosis regulatory molecules and immunocytes. Red indicated positive correlations, and blue indicated negative correlations. Asterisks denoted p-value (**p < 0.01, *p < 0.05). Blank cells represented no statistical significance of the correlation.

Since cancerous immunity plays a crucial role in tumor progression and cell proliferation, we speculated that the tumor immune microenvironment in different clustering subtypes might be distinctive. We first investigated the correlations between necroptosis phenotypes and multiple immunomodulators and immune checkpoints. The results of necroptosis influences on the tumor immune microenvironment showed that significantly up-regulated expression levels of chemokines existed in Clusters B and C, such as CCL11, CCR1, CXCL10, and PPBP (**Figure 3A**, upper part). The increasing expressions of interleukins, interferons, receptors, and some other cytokines were also seen in Clusters B and C, whereas Cluster A had strongly down-regulated levels of the immune molecules (**Figure 3A**, middle and bottom parts). Compared to Clusters A and B, Cluster C exhibited the highest expressions of the seven immune molecules that demonstrate underlying targets for immunotherapy, including CD274 (PD-L1), PDCD1 (PD-1), CD247, PDCD1LG2 (PD-L2), CTLA-4, TNFRSF9, and TNFRSF4 (**Figure 3B**).

We further surveyed the infiltration levels of 20 immune cell types and the levels of each step in immune cycles. The exploration of gene signatures implied that immunocyte infiltration was dramatically higher in Cluster B followed by Cluster C, while it exerted significantly lower fractions in Cluster A, such as CD4^+^ naïve T cells, follicular helper T cells, and M1 macrophages (**Figure 3B**). It was noteworthy that activated memory CD4^+^ T cells had the lowest abundance in Cluster B but a higher level in Clusters A and C. The scores of seven immune cycle signals defined by the events of anti-tumor immune response happening in each step to exterminate tumor cells were calculated by the ssGSEA algorithm. All steps showed higher scores in Clusters B and C but showed particularly low levels in Cluster A (**Figure 3B**). Moreover, Cluster A also had significant weakness of MeTIL, which suggested a low fraction of tumor-infiltrating immune cells (**Figure 3B**).

The TIDE algorithm and the ESTIMATE method were also adopted to compare the heterogeneity between immune and matrix components in immune subtypes of TME. Thus, the correlation between necroptosis fractions and potentially immunotherapeutic efficacy could be quantified. It was found that Cluster B had the highest TIDE and dysfunction score, followed by Cluster C, and then Cluster A, whereas, an opposite result was found in the exclusion analysis (p < 0.001, **Figure 3B** upper part**;** **Supplementary Figure 4A-C**). Clusters B and C had significantly higher immune and stromal scores than Cluster A, suggesting Cluster A showed the highest tumor purity (p < 0.001, **Figure 3B** upper part**;** **Supplementary Figure 4D-F**). Additionally, we investigated biomarkers of several immune pathways. It was shown that significantly higher normalized enrichment scores of functions were mainly located in Cluster C, including co-inhibition of APC, checkpoint, inflammation-promoting, MHC class I, para-inflammation, co-inhibition/stimulation of T cells, and type I IFN response (all p < 0.001, **Figure 3C**). Cluster B showed relatively higher scores of co-stimulation of APC, CCR, cytolytic activity, HLA and type II IFN response, while the lowest abundance was observed in Cluster A (all p < 0.001, **Figure 3C**). Through a correlation strategy, several necroptosis regulatory genes, including ALK, AXL, KLF9, LEF1, TNFRSF1B, and ZBP1, were positively associated with most of the immunocytes, while negative correlations were demonstrated in molecules such as BNIP3, DIABLO, FADD, IDH1, IDH2, PLK1, and SLC39A7 (**Figure 3D**).

### Delineation of Genome Alterations Among Necroptosis Phenotypes

**Figure 4:**
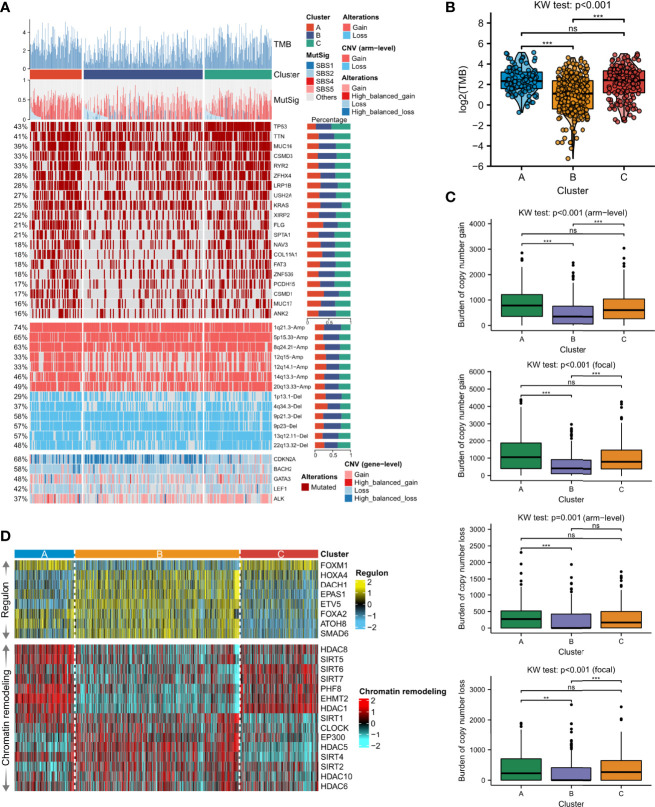
Distinction of the genomic features among the three necroptosis phenotypes. **(A)** Somatic mutation landscape according to necroptosis clustering subtypes. Tumor mutation burden (TMB), the relative contribution of the four mutational signatures (SBS1, SBS2, SBS4, and SBS5), selected top-mutated genes, and top-broad-level copy number alterations (q-value<0.05), and selected necroptosis molecules were shown from the top to the bottom panels. The proportion of variation conversions was presented in the right stacked bar plots. **(B)** TMB differences among the three clusters were revealed by Kruskal-Wallis test. **(C)** Focal and arm-level copy number variations among the necroptosis phenotypes were revealed by Kruskal-Wallis test. Asterisks denoted p-value (***p<0.001, **p<0.01 and ns was the abbreviation of no significance). **(D)** The heatmap showed regulon activity profiles for eight transcription factors (upper panel) and potential regulators associated with chromatin remodeling (bottom panel).

We further explored the genomic differences of the identified LUAD molecular phenotype, including mutation signature, SNV, and the activity of the transcription factor regulatory network. It was found that Cluster A and Cluster C showed significantly higher TMB (p < 0.001, **Figure 4A, B**) than Cluster B. We inferred that four mutational signatures were related to LUAD, namely SBS1 (age-associated), SBS2 (APOBEC activity-associated), SBS4 (smoking-associated) and SBS5 (ERCC2 mutation-associated). However, statistical significance was only detected in SBS1 showing that Cluster B had more mutations in age-associated signatures compared to Cluster A (p < 0.001, **Figure 4A** **;** **Supplementary Figure 5A**), and that Cluster A showed a higher frequency of mutations in smoking-associated signatures compared to Cluster C, followed by Cluster B (p < 0.001, **Figure 4A** **;** **Supplementary Figure 5B**). Therefore, these mutation signatures can serve as the explanation of perturbation of each molecular necroptosis phenotype (24). Of the top-20 frequently mutated genes, we found that Cluster C was more enriched in mutations of TP53, TTN, MUC16, CSMD3, and RYR2, and Cluster B demonstrated a relatively lower mutation frequency compared to Clusters A and C (**Figure 4A**). The burden of copy number amplifications and deletions fell in Clusters B and C but significantly increased in Cluster A at both the focal and arm-level, and Cluster B presented a dramatically lower CNV burden (**Figure 4C**). The frequency of CNV and GISTIC score distributions in different chromosomes was explored, verifying our results and showing that the most extreme frequency difference between gain and loss was located on chromosome 8 and 14 (**Supplementary Figure 6A, B**).

To further analyze transcriptomic differences among the clusters, a total of 8 LUAD-specific transcription factors and 15 underlying regulators correlated to cancer chromatin remodeling were surveyed. We confirmed the biological pertinence of the three necroptosis clusters since regulon activity was closely connected with the clusters (**Figure 4D**, upper part). Clusters A and C shared similar patterns of regulon activity, whereas Cluster A differed with higher activity of FOXA2 and ATOH8, while Cluster C was distinctly correlated with activated HOXA4. Cluster B contained a high concentration of regulons such as HOXA4, DACH1, EPAS1, ETV5, FOXA2, ATOH8, and SMAD6. Regulon activity information related to cancerous chromatin remodeling underlined alternative doable differential regulative patterns among the three necroptosis phenotypes, suggesting that epigenetically-driven transcriptional networks were, perhaps, important differentiators of these clusters (**Figure 4D**, bottom part). We found that Clusters A and C also shared similar regulon patterns, whereas Cluster A exhibited higher activity of SIRT1, SIRT4, and SIRT5. Meanwhile, regulons of SIRT1, CLOCK, EP300, HDAC5, SIRT4, SIRT2, HDAC10, and HDAC8 were significantly enriched in Cluster B.

### Dysfunction of Signaling Pathways

**Figure 5:**
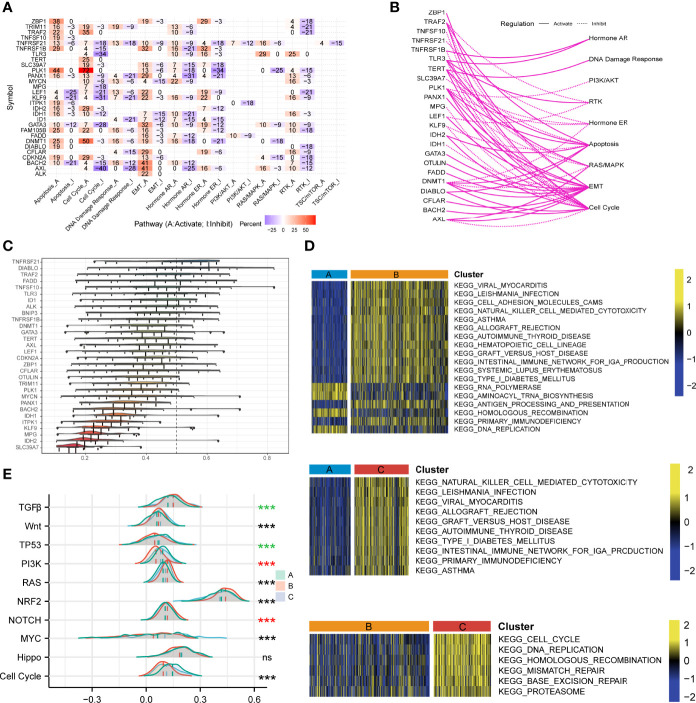
Investigations of necroptosis- and cancer-related signaling pathways. **(A)** The heatmap illustrated the correlations between the mRNA expressions of the 29 necroptosis molecules in universally recognized cancer-related signaling pathways. Only a function (inhibited or activated) in at least 5 cancer types was displayed. “Pathway activate” in red represented the percentage of cancers where a pathway might be activated by the necroptosis regulators, and inhibition in a similar way illustrated as “Pathway inhibit” in blue. **(B)** The network showed the relationship between necroptosis regulatory genes by a line connection. The solid line meant activation, while dashed lines meant inhibition. **(C)** The distributions of functional similarities of the necroptosis-related molecules were summarized as complex boxplots. The boxes represent the middle 50% of the similarities, and the upper and bottom boundaries showed the 75th and 25th percentile. The lines in the boxes indicated the median values of the functional similarities. The continuous distribution was shown as the lines on boxes. The dots represented raw data. **(D)** Gene set variation analysis enrichment demonstrated the activated and inhibited states of biological pathways in distinct necroptosis regulatory phenotypes. The heatmap was employed to visualize the activity of biological pathways. Yellow represented activated pathways, and blue represented inhibited pathways. **(E)** The mountain graph showed the ten vital well-known cancerous signaling pathways among the three necroptosis clustering subtypes. Asterisks denoted p-value (***p < 0.001 and ns was the abbreviation of no significance), the color of which represented different statistical test methods. Black indicated Kruskal-Wallis test; red indicated one-way ANOVA test, and green indicated Welch one-way ANOVA test.

We explored the differences in gene expression among the three phenotypes of necroptosis and analyzed the characteristics of several cancer-related signaling pathways, which help to understand potentially latent regulatory mechanisms and druggable pathways. Necroptosis regulatory molecules were associated with activated pathways, such as apoptosis, cell cycle A, EMT-A, and hormone AR-A, but consistently inhibited functions such as cell cycle I, hormone AR/ER-I, and RTK-I in pan-cancer (**Figure 5A**). In LUAD, most necroptosis-related genes activated pathways such as the cell cycle, EMT, and apoptosis, whereas they inhibited hormone ER and RTK signals (**Figure 5B**). We used semantic similarity to aggregate tightly related categories to screen for hub genes among the 30 regulators playing a part in necroptosis. The result indicated that TNFRSF21 showed the highest weight, followed by DIABLO and TRAF2 (**Figure 5C**). To analyze the biological behaviors of these distinct necroptosis phenotypes, we conducted GSVA enrichment explorations. As illustrated in **Figure 5D**, Cluster A was markedly enriched in RNA polymerase, aminoacyl tRNA biosynthesis, homologous recombination, and DNA replication. Cluster B included biological pathways associated with viral myocarditis, cell adhesion molecules (CAMs), natural killer cell-mediated cytotoxicity, and hematopoietic cell lineage. Besides, Cluster C was prominently enriched in primary immunodeficiency, the intestinal immune network for IGA production, cell cycle, DNA replication, mismatch repair, and base excision repair. We also assessed other carcinopathways in different clustering subtypes. The scores of NOTCH, NRF2, RAS, and TGFβ were significantly high in Cluster B, while cell cycle and TP53 signaling pathways were activated in Cluster C (**Figure 5E**). Moreover, the association between the three phenotypes and the cGAS-STING signaling pathway was examined. It demonstrated that cGAS (p < 0.001), IRF3 (p < 0.001), and TBK1 (p = 0.001) had the highest expression levels in Cluster C but were most down-regulated in Cluster A (**Supplementary Figure 7A-D**), which implied the significance of necroptosis in shaping the tumor immune microenvironment and immune response and further suggested the potential viability of necroptosis in chemotherapy and immunotherapy for LUAD treatment.

### Clinical Values of the NecroScore

**Figure 6:**
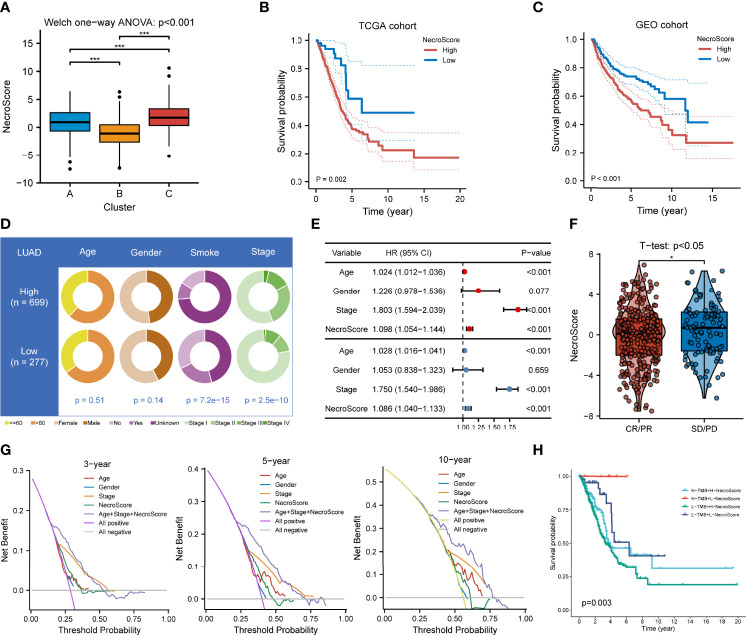
Clinical significance of NecroScore. **(A).** A Welch one-way ANOVA test confirmed the NecroScore at three distinct levels of necroptosis. The line in the box represents the median value, and the black dots represented outliers. Asterisks indicated p-value (***p<0.001). **(B, C).** Kaplan-Meier curves and log-rank test showed significant differences of overall survival probability between the low- and high-NecroScore groups in the TCGA-LUAD and GEO-meta cohorts, respectively. **(D).** The pie chart showed variations of clinicopathologic characteristics of lung adenocarcinoma between the low-and high-NecroScore groups by Fisher’s exact test. **(E).** A univariate (upper part) and multivariate (lower part) Cox model showed the independent prognostic values of age, pathologic stage, and the NecroScore shown by a forest plot. **(F).** Distribution of the NecroScore in distinct groups after primary therapy response group (CR, complete response; PR, partial response; SD, stable disease; PD, progressive disease) was revealed by t-test. **(G).** Decision analysis showed the clinical efficiency of the NecroScore. The x-axis represented the threshold probability threshold, and the y-axis represented the net benefit of 3, 5, and 10 years. The combination of age, pathologic stage, and the NecroScore exhibited the highest net benefit stably. **(H)**. Survival analysis for patients with different tumor mutation burden and NecroScore stratifications was performed by log-rank test and visualized by Kaplan-Meier curves. “H” represented high and “L” represented low.

We then developed a scoring system named NecroScore based on the molecules related to necroptosis to measure the level of necroptosis in LUAD patients. Patients were classified into low-and high-NecroScore groups at the optimal cutoff in TCGA and GEO-meta cohorts independently, where the value of TCGA-LUAD was determined at -3.80 and that of GEO was determined at -0.22 (**Supplementary Table 4**). By comparing NecroScore levels among the three clustering subtypes, it was shown that NecroScore was the highest in Cluster C, followed by Cluster A, and then Cluster B by the Welch one-way ANOVA test (**Figure 6A**). Patients with a higher NecroScore experienced a worse prognosis than those with a lower NecroScore according to KM survival analysis in both the TCGA (p = 0.002, **Figure 6B**) and the GEO cohort (p < 0.001, **Figure 6C**). Not only that, patients of different clinical stratifications, including young and old, women and men, and early-stage and advanced-stage also had survival significance (**Supplementary Figure 8**). Higher non-smoker rates (p < 0.001) and more patients in advanced stages (p < 0.001) were found in the high-NecroScore group, while age and gender distribution had no statistical difference (**Figure 6D**). A univariate Cox model showed age, pathologic stage, and NecroScore were significantly associated with survival outcomes (**Figure 6E**, upper part), and a multivariate Cox regression verified that age (HR = 1.028, 95%CI = 1.016-1.041, p < 0.001), stage (HR = 1.750, 95%CI = 1.540-1.986, p < 0.001) and NecroScore (HR = 1.086, 95%CI = 1.040-1.133, p < 0.001) could serve as independent prognostic factors (**Figure 6E**, bottom part). The significant therapeutic advantages in patients with low NecroScore compared to those with high NecroScore were determined by t-test (p<0.05, **Figure 6F**). Among the three independent prognostic factors, the stage exhibited the highest net benefit, followed by age, and then NecroScore (**Figure 6G**). Moreover, the combination of the three variables had the highest level of net benefit according to decision analysis in 3, 5, and 10 years (**Figure 6G**). A nomogram model was established to predict the 1-, 3- and 5-year survival of LUAD patients (**Supplementary Figure 9A**). Calibration curves validated that the nomogram model had optimistic predictions for overall survival outcomes (**Supplementary Figure 9B**). We also found that patients with a combination of low TMB and high NecroScore had a poor prognosis, while those with high TMB and low NecroScore demonstrated an obvious advantage (**Figure 6H**).

We further investigated the correlations between immunocyte infiltrating levels. The heatmap of immune responses based on TIMER, CIBERSORT, CIBERSORT-ABS, ESTIMATE, QUANTISEQ, MCP-counter, and XCELL using the ssGSEA method is illustrated in **Supplementary Figure 10A**. It shows that the infiltrating abundance of several cell types is negatively associated with NecroScore, such as myeloid dendritic cells, cancer-associated fibroblasts, hematopoietic stem cells, and monocytes. Different gene markers of immune cells, collected from another study, were used to validate the result. Most of the signs presented negative correlations, especially in endothelial cells, resting dendritic cells, and resting mast cells, and weak associations could be found in activated memory CD4^+^ T cells and fibroblasts (**Supplementary Figure 10B**). As to immunophenotypes, a higher proportion of C1 (20%h vs. 6%l), C2 (34%h vs. 19%l), and C6 (6%h vs. 2%l) were in the high-NecroScore group, while more samples of C3 (36%h vs. 66%l) and C4 (4%h vs. 6%l) were located in the low-NecroScore group (**Supplementary Figure 10C**). The overall distribution exerted significance as tested by Fisher’s exact test (p = 0.001). Evidence from TCIA revealed that low-NecroScore patients had significantly lower IPS of positive or negative status of anti-CTLA-4 with no response to PD-1 (**Supplementary Figure 10D**), suggesting anti-CTLA-4 inhibitors might also have effects on negative PD-1 status patients with low scores. Moreover, we found that several immune checkpoints, such as CD274, PDCD1, PDCD1LG2, LAG-3, and TMIGD2, were down-regulated in the low-NecroScore group (**Supplementary Figure 11A**). Interestingly, the TIDE score, MSI, and dysfunction scores were significantly increased in the low-NecroScore group. At the same time, low NecroScore patients had lower exclusion scores (**Supplementary Figure 11B**), indicating that individuals with negative or weak-expressed immune checkpoints might also demonstrate promising results from immune checkpoint blockade (ICB) therapy.

### Mutation Status in the Low- and High-NecroScore Groups

To analyze the NecroScore-related mechanisms in LUAD, somatic mutations of the TCGA-LUAD cohort were surveyed. Comparing the mutation frequency between the two scoring groups, more mutations were found in the high-NecroScore group (r = 0.3, p < 0.001; **Supplementary Figure 12A**), including non-synonymous (r = 0.29, p < 0.001) and synonymous mutations (r = 0.3, p < 0.001; **Supplementary Figure 12B, C**). Meanwhile, a significantly higher mutant frequency of TP53, TTN, PCDH15, LRP1B, ZFHX4, NALCN, CSMD3, MUC16, XIRP2, APOB, PAPPA2, PRDM9, ANK2, and LRRC7 was observed in the high-NecroScore group, while KEAP1 had more mutations in the low-NecroScore group, shown by a forest plot (**Supplementary Figure 12D**). In addition, significant co-occurrences existed among these genes (**Supplementary Figure 12E**). We also uncovered the influence of high mutation genetic variation on the NecroScore and found that the NecroScore was positively correlated with the necroptosis-related gene CDKN2A mutant frequency (**Supplementary Figure 12F**).

### Drug Discovery and Chemotherapeutic Response Analysis

**Figure 7:**
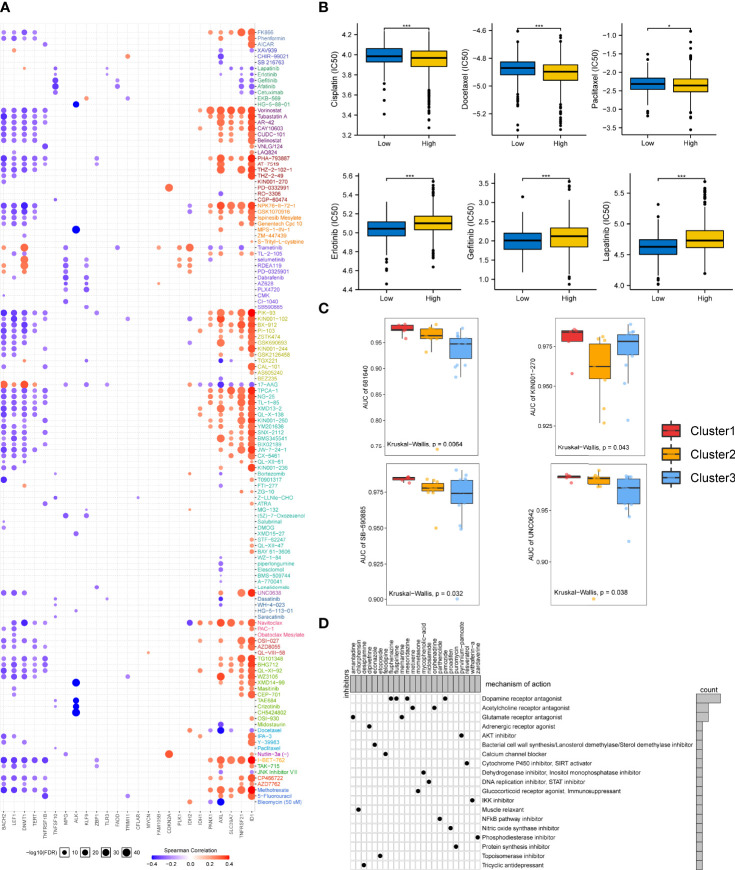
Drug sensitivity in necroptosis-based subtypes and correlation between chemotherapeutic potential and the NecroScore. **(A)**. The bubble diagram illustrated the results of correlation analysis of the necroptosis regulatory molecules on resistance to common clinical chemotherapeutics. Red indicated positive correlations, meaning that overexpressed mRNA levels of the necroptosis-related molecules were resistant to the drug and vice versa. The darker the color, the larger the correlation coefficients. The bubble size denoted the negative value of log10 of false discovery rate (FDR). **(B)**. A Wilcoxon test showed the differences in half-maximal inhibitory concentration (IC50) between the low- and high-NecroScore groups. Asterisks indicated p-value (***p < 0.001, *p < 0.05). **(C)**. Differences of the area under the curves (AUCs) of drug response among the three lung adenocarcinoma cell line clustering subtypes were revealed by Kruskal-Wallis test. **(D)**. The heatmap showed each potential compounds sharing mechanisms of action (MoA, in rows) and sorted by descending number of compound with shared MoA shown in the right bar plot.

We analyzed the association between necroptosis-related molecules and the clinical efficiency of LUAD treatment. According to the drug response data from GDSC, it was shown that some genes, such as BAHC2, LEF1, DNMT1, TERT, TNFRSF1B, TNFSF10, MPG, and ALK have synergistic effects with drugs, whereas PANX1, AXL, SLC39A7, TNFRSF21, and ID1 interacted antagonistically with drugs (**Figure 7A**). ALK manifested a significantly synergistic interplay with CH5424802 (alectinib, ALK, and RET inhibitor, **Figure 7A**). It was also evident that ID1 had the strongest negative correlation with drugs such as vorinostat (HDAC inhibitor), PHA-793887 (CDK2 inhibitor), PIK-93 (PI4K inhibitor), and SNX-2112 (HSP90 inhibitor, **Figure 7A**).

Given the importance of necroptosis regulatory molecules in chemotherapy, we further analyzed whether the NecroScore could accurately predict the chemotherapeutic sensitivity of LUAD patients. Ridge regression was utilized to predict IC50 in the low-and high-NecroScore groups. Results showed that higher NecroScore had lower IC50 of several LUAD-specific drugs such as cisplatin, docetaxel, and paclitaxel. In contrast, patients with a low NecroScore might respond more sensitively to erlotinib (tyrosine kinase inhibitor/TKI), gefitinib (EGFR-TK inhibitor), and lapatinib (TKI) (**Figure 7B**), suggesting that chemotherapy was a promising option for the high-NecroScore group. In contrast, targeted therapy for the low-NecroScore group might lead to favorable outcomes. The AUC of drug responses within cell line clusters obtained from the classifier based on the necroptosis phenotypes was compared. Only 274 compounds tested on over 90% of the LUAD cell lines were employed for analysis (**Supplementary Table 5** **-** **7**). AUCs of SB-590885, a B-RAF inhibitor, and UNC0642, a G9a/GLP inhibitor, were significantly lower in the Cluster 3 LUAD cell lines, but the information from 681640 and KN001-27 was absent to our knowledge (**Figure 7C**).

Furthermore, we identified potential compounds that could target stemness features and the NecroScore difference. Analysis of 24 compounds revealed 19 MoA was shared by these compounds (**Figure 7D**). Four compounds (fluphenazine, fluspirilene, mesoridazine, and pimozide) shared the MoA of dopamine receptor antagonist; metixene and orphenadrine shared the MoA of acetylcholine receptor antagonist; and amantadine and memantine shared the MoA of glutamate receptor antagonist. It could also be seen that pyrvinium-pamoate presented the MoA of the AKT inhibitor. The results help to adopt more tailored chemotherapeutic strategy for each stratified LUAD group based on the NecroScore, coupled with a reference to the mutation characteristics (**Supplementary Figure 12**).

## Discussion

In this study, we showed the transcriptional variations of 30 necroptosis regulatory molecules in LUAD, revealing that the heterogeneity of necroptosis-related gene expression was associated with genome changes. Regulating the expression levels of necroptosis molecules may influence genome instability of LUAD cells (25), which can be utilized to seek chemotherapeutic targets inducing necroptosis. A total of 1,064 LUAD patients were distinguished into three molecular phenotypes based on the necroptosis molecule expression levels. The significant differences were confirmed in clinicopathologic features and TIME among distinct clustering subtypes. A previous study verified that targeting necroptosis could induce the innate immune system to kill neoplastic cells by activating RIPK1 and RIPK3 (26), the mutation of which exists in multiple tumors (27). Our results implied a significant interrelationship between necroptosis levels and TIME.

Changes in mRNA transcriptional levels among different necroptosis phenotypes determined that clustering subtypes were correlated with specific cancer-related pathways and potential immunotherapeutic efficiency. Cluster A was characterized by immune suppression of TIME and high tumor purity, presenting a cold tumor-like phenotype. Cluster B was characterized by several immune cells, low overall mutational load, upregulated CXCL12 expression, and TGFβ and Wnt signaling pathway activation. Cluster C was characterized by activating immunocyte infiltration and immune-related pathways such as natural killer cell-mediated cytotoxicity, primary immunodeficiency, mismatch repair, and high somatic mutations. These two types present the features of hot tumors. We also found a significant correlation between the cGAS-STING signaling pathway and necroptosis subtypes. Activating the pathway can detect cytoplasmic DNA, trigger immune effects, and make the tumor cells susceptible to apoptosis (28, 29) and thus, provide a potential therapeutic approach for patients of Cluster A. The corresponding result validated our previous conclusion that Cluster C, indeed, had more fractions of anti-tumor immunity in LUAD. Our study also found a strong correlation between necroptosis and the expressions of chemokines and receptors. Among them, CCL5 and CXCL9 have been widely investigated because their overexpressed levels were correlated with CD8^+^ T cell infiltration in cancers, namely immunoreactive tumors (30), which supported our results that abundant immune infiltration was enriched in Cluster C. Moreover, targeting CCL5 to augment TGFβ signaling, which was inhibited in Cluster C, is an alternative treatment protocol (31). The highest immune checkpoint expressions were observed in Cluster C, followed by Cluster B, and then immune-cold Cluster A. Patients with upregulated PD-1/PD-L1 respond better to ICI treatment in general (32). Therefore, it is possible to turn Cluster A into a “hot” tumor sensitive to anti-PD-1/PD-L1 ICI by further increasing its Hippo signaling level (33). In addition, regulon activity based on transcriptional factors and chromatin remodeling showed tight correlations with necroptosis phenotypes, supporting the regulators’ roles as hub drivers of LUAD (34, 35) and providing underlying interventional targets used for phenotype classification and therapy adoption (36). Combining the results above and drug analysis in the study, it could be speculated that the combination of ICI and necroptosis inducers might show promising therapeutic outcomes for LUAD, and this study would help in the development of novel combination treatment techniques as well as new immunotherapeutic drugs in the near future.

Given the critical function of necroptosis in immune control and the variability of necroptosis subtypes of LUAD, it is critical to characterize the expression of necroptosis regulatory factors in LUAD patients. To this end, we constructed a quantified prognostic system named NecroScore to assess the necroptosis molecules in LUAD patients. The NecroScore was reliable for evaluating patients’ survival outcomes. Patients from the high-NecroScore group had higher expressions of immune checkpoints, whereas, it was found that there was a negative correlation between the NecroScore and infiltrating levels of anti-tumor immunocytes and lower TIDE scores in the low-NecroScore group. A recent phase-3 clinical trial revealed that pembrolizumab (anti-PD-1/PD-L1), plus pemetrexed and platinum, enhanced the survival of LUAD patients despite PD-L1 expression (37). Hence, it favored that patients with a lower NecroScore might benefit from ICI treatment, such as those exhibiting characteristics of necroptotic Cluster B identified in this study. In contrast, patients from the high-NecroScore group with higher TIDE scores and lower MSI might have low ICI responses for immune evasion by T-cell exclusion (38). Above results suggest NecroScore may act as a preferable candidate tool for genomic aberrations and traditional histopathologic index.

We also found that high-NecroScore patients had more mutations such as TP53, TTN, and PCDH15. It has been reported that nonsense or truncating TP53 mutations are correlated with poor prognosis during immunotherapy (39, 40). However, TP53 mutations, with wild types of STK11 and EGFR, identify high CD8^+^ T-cell density and PD-1 expression (41). Genome editing technologies such as CRISPR/Cas9 can be employed to alter mutation profiles and provide a prerequisite for effective immunotherapy. Further explorations are still needed to confirm more detailed mutational features of the low- and high-NecroScore groups to tailor immunotherapeutic plans. The investigation of MoA led us to potential candidate targets for different therapies of distinct scoring subtypes (42). Multiple lines of evidence have validated that necroptosis-related genes and pathways play a crucial role in chemotherapy (43–45). This study found considerable discrepancies between chemotherapeutic drugs and their corresponding mRNA target expressions, and the NecroScore could effectively contribute to the application of chemotherapies. Our results have implications for clinical practice. The NecroScore could be employed to estimate the expression features of necroptosis-associated genes and the immunocyte infiltration of LUAD TIME, confirming the molecular subtypes or immunophenotypes of LUAD, and predict the survival of and medication treatment efficiency for patients.

However, there are several shortcomings in the present study. Firstly, this study was retrospective and the data was only collected from online databases. Although this research conducted batch effect correction, to a great extent, for thousands of patients, effects for genome analysis were still on, and further investigation should be conducted using larger data sets from multicenter. Secondly, the molecular stratifications were only based on transcriptional expressions, mutational, metabolic, proteomic, and even imaging characteristics should be integrated to reveal comprehensive heterogeneity among distinct subtypes. We also expect to use single-cell sequencing technology to unmask cell fractions and genomic distinctions among the phenotypes. Moreover, wet-lab experiments *in vivo* and *in vitro* (i.e., flow cytometry) are required to verify the roles of necroptosis regulatory molecules in LUAD and hopefully to interrogate the impact of single nucleotide variants and copy number alterations on necroptosis.

## Conclusions

This study depicted the molecular and immune landscape of necroptosis molecules in the LUAD tumor microenvironment. The three necroptosis phenotypes could distinguish the clinical features, immune infiltration, and somatic mutational characteristics of LUAD. To some extent, the developed NecroScore system could predict prognosis and chemotherapeutic treatment efficiency. Our comprehensive evaluation of individual tumor necroptosis patterns with multi-omic approaches will deepen our understanding of the tumor immune microenvironment and tailor more effective immunotherapy strategies.

## Data Availability Statement

All data used in this work can be acquired from (TCGA,(https://portal.gdc.cancer.gov/), GEO (https://www.ncbi.nlm.nih.gov/geo/), UCSC Xena (https://xenabrowser.net/datapages/), GDSC (https://www.cancerrxgene.org/), TIMER (https://cistrome.shinyapps.io/timer/), TIP (http://biocc.hrbmu.edu.cn/TIP/index.jsp), MSigDB (http://www.gsea-msigdb.org/gsea/index.jsp), TCIA (https://tcia.at/) and CLUE (https://clue.io/).

## Author Contributions

CZ and XL designed the study. KX and ZJ collected study data and performed computational analysis. CZ, KX and AA wrote the manuscript draft. AA edited the manuscript draft and did subsequent revision. All authors have read and approved the manuscript. CZ and KX have contributed equally to this work and share first authorship.

## Funding

This research was supported by grants No.2014CFB394, 2019CFB721 from the Natural Science Foundation of Hubei Province (CN) and No.WJ2017M027 from Health and Family Planning Commission of Hubei Province (CN), No.Y-HS202101- 0079 from Cisco hausen Cancer Research Foundation.

## Conflict of Interest

The authors declare that the research was conducted in the absence of any commercial or financial relationships that could be construed as a potential conflict of interest.

## Publisher’s Note

All claims expressed in this article are solely those of the authors and do not necessarily represent those of their affiliated organizations, or those of the publisher, the editors and the reviewers. Any product that may be evaluated in this article, or claim that may be made by its manufacturer, is not guaranteed or endorsed by the publisher.
