# Supplementary material for: Necroptosis Identifies Novel Molecular Phenotypes and Influences Tumor Immune Microenvironment of Lung Adenocarcinoma
Source: Front Immunol. 2022 Jul 14;13:934494. doi: 10.3389/fimmu.2022.934494 (PMC9331758; doi:10.3389/fimmu.2022.934494)
Supplement: Supplementary file 1 [file DataSheet_1.docx]

**Supplementary Descriptions**

**Supplementary Table 1.** The transcriptional changes of the 30 necroptosis molecules in lung adenocarcinoma.

**Supplementary Table 2.** Molecular classification of lung adenocarcinoma based on necroptosis regulatory genes.

**Supplementary Table 3.** Gene markers of several cancer-related pathways.

**Supplementary Table 4.** The NecroScore and its stratifications of lung adenocarcinoma patients.

**Supplementary Table 5.** Expression levels of differentially expressed necroptosis molecules in lung adenocarcinoma cell lines.

**Supplementary Table 6.** Area under the curves (AUCs) of 274 drugs in lung adenocarcinoma cell lines.

**Supplementary Table 7.** Clustering subtypes of samples in lung adenocarcinoma cell lines.

**Supplementary Figure 1.** **The workflow of the study.**

**Supplementary Figure 2. Necroptosis regulatory molecules and clinical features of lung adenocarcinoma.**

**A.** The expression variations of the necroptosis-related genes between adjacent and tumor tissues in the TCGA-LUAD and GTEx datasets. The line in the box represented the median value. The upper and bottom boundaries showed the 75th and 25th percentile. Asterisks indicated p-value (***p<0.001 and ns was the abbreviation of no significance). **B.** Significant differentially expressed necroptosis-related genes among distinct pathologic stages were examined by Kruskal-Wallis test. **C.** Using Pearson correlation and hierarchical clustering, the 29 necroptosis regulatory molecules were divided into four clusters represented by multiple colors. The line linking each two cells represented the interplay between the two factors. Red indicated a positive correlation, and light blue indicated a negative correlation. The thicker the line, the stronger the correlation. The size of the cells represented the effects on overall survival (OS) probability estimated by log-rank test, and p-value was transformed by –log10. The points in the circle represented the prognostic role quantified by a univariate Cox proportional hazard regression. Green denoted risk factors for OS, namely the hazard ratio was over 1 with p<0.05. Light purple denoted favorable factors for OS, namely the hazard ratio was less than 1 with p<0.05. **D.** A univariate Cox model revealed the prognostic features of necroptosis-related genes in the TCGA-LUAD cohort by a forest plot. Red indicated significant risk variables, and green indicated significant favorable variables.

**Supplementary Figure 3. Track of the unsupervised K-means-based consensus-clustering.**

**A.** The heatmap showed the distribution of 2, 3 and 4 clusters respectively. **B.** The line graph showed the delta area of 2, 3 and 4 clusters. **C.** The consensus cumulative distribution function of different clusters. The minimum of distance between the upper line and the lower line determined the optimal clusters.

**Supplementary Figure 4. Quantified tumor immune microenvironment.**

**A-C.** Boxplots showed the differences of TIDE, dysfunction and exclusion scores among the necroptosis clusters. The points represented the raw data distribution. The line in the box represented the median value. **D-G.** Boxplots showed the differences of tumor purity, immune and stromal scores among the necroptosis clusters. The points represented the raw data distribution. The line in the box represented the median value.

**Supplementary Figure 5.** **Mutational signature differences among the clusters of SBS1 (A) and SBS4 (B) were revealed by KW test.**

**Supplementary Figure 6. Copy number variation (CNV) distribution in different chromosomes.**

**A-B.** The plot illustrated the copy number frequency in each cluster respectively. Red represented CNV amplifications, and blue represented CNV deletions. Gene segments were placed according to their location in chromosomes, ranging from chromosomes 1 to 22.

**Supplementary Figure 7. The levels of cGAS-STING-related genes among the three necroptosis phenotypes examined by Kruskal-Wallis and Welch one-way ANOVA tests.**

Upper and lower ends represent the quartile range of the value. The line in the box represented the median value. The points represented the raw data distribution. Asterisks indicated p-value (***p<0.001, **p<0.01 and ns was the abbreviation of no significance).

**Supplementary Figure 8.** **Survival differences between the low- and high-NecroScore groups.**

The survival differences in distinct clinicopathologic stratifications of age (**A**), gender (**B**) and pathologic stage (**C**) were revealed by log-rank test between the low- and high- NecroScore groups.

**Supplementary Figure 9. A combined diagnostic model.**

**A.** A nomogram model developed with age, gender, pathologic stage and the NecroScore. An example was illustrated for prediction shown in highlighted lines and points. Asterisks indicated p-value (***p<0.001). **B.** 1-, 3- and 5-year calibration curves were plotted to validate the effectiveness of the nomogram model. The blue crosses represented the results of each point after stratified Kaplan-Meier corrections. The gray vertical lines stuck at the top represented the distribution of survival probability of the prediction.

**Supplementary Figure 10. The correlation between immune characteristics and the NecroScore.**

**A-B.** The thermogram and the lollipop showed the associations between the NecroScore and immunocyte infiltrating levels by different methods. **C.** Fisher’s exact test demonstrated the significantly different distributions of immune subtypes in the low- and high-NecroScore groups. **D.** Immunophenoscore (IPS) to predict anti-CTLA-4 and anti-PD-1 differences between the low- and high-NecroScore groups were showed by violin plots. The horizontal line in the violin represented the median value. The length of vertical line in the violin represented 95% confidence interval. And the length of each violin indicated the raw data distribution. Asterisks indicated p-value (***p<0.001 and ns was the abbreviation of no significance).

**Supplementary Figure 11. Immune checkpoint inhibitor (ICI) therapy for the low- and high-NecroScore groups.**

**A.** The expression changes of several immune checkpoints between the two NecroScore subtypes by Wilcoxon rank-sum test. **B.** Predicted efficiency of ICI therapy for the low- and high-NecroScore groups by Wilcoxon rank sum test and t-test. Asterisks indicated p-value (***p<0.001). The line in the box represented the median score, and the black dots represented outliers.

**Supplementary Figure 12. The NecroScore was correlated to tumor mutation status.**

**A-C.** The relationship between non-synonymous, synonymous, all mutation counts and the NecroScore in the low- and high-NecroScore groups. Blue represented low-NecroScore samples, and grey represented high-NecroScore samples. **D.** A forest plot showed differentially mutated genes in lung adenocarcinoma patients of the low- and high-NecroScore groups. **E.** Interplay effects of differentially mutated genes in lung adenocarcinoma patients including co-occurrence and mutual exclusion. **F.** The correlation between the NecroScore and mutations of necroptosis-related molecules in lung adenocarcinoma revealed by permutation test. The histogram represented the NecroScore, each column represents individual patient. Samples were ordered by the NecroScore level. Red indicated mutation and gray indicated no mutation.
