# Supplementary material for: Necroptosis Identifies Novel Molecular Phenotypes and Influences Tumor Immune Microenvironment of Lung Adenocarcinoma
Source: Front Immunol. 2022 Jul 14;13:934494. doi: 10.3389/fimmu.2022.934494 (PMC9331758; doi:10.3389/fimmu.2022.934494)
Supplement: Supplementary file 2 [file DataSheet_2.docx]

**Supplementary Materials and Methods**

**Data sources and preprocessing**

The LUAD whole genome-wide expression profiles in “fragments per kilobase per million (FPKM)” and “counts” format, clinical annotations and simple nucleotide variation (SNV) estimated by “VarScan2 Variant Aggregation and Masking” tool were retrospectively downloaded from The Cancer Genome Atlas (TCGA, https://portal.gdc.cancer.gov/) and Gene Expression Omnibus (GEO, https://www.ncbi.nlm.nih.gov/geo/) database. A total of 1064 patients were enrolled for analysis, including those from TCGA-LUAD (n=513), GSE13213-GPL6480 (n=117) (1), GSE26939-GPL9053 (n=116) (2), GSE29016-GPL6947 (n=72) (3) and GSE31210-GPL570 (n=246) (4, 5). A logarithm of base 2 and “transcripts per kilobase per million (TPM)” transformation were applied to the gene-level FPKM data of TCGA cohort since the format aligns more closely with the definition of relevant expression levels. Differential expression analysis of necroptosis-related molecules in LUAD based on Toil-processed (6) data and GISTIC copy number variation (CNV) information of 530 samples measured by Illumina were retrieved from UCSC XENA (https://xenabrowser.net/datapages/). Batch effects from non-biological technical biases were corrected using the ComBat method of the R package “sva”. Principle component analysis (PCA) was employed to examine the correction degree. Reverse phase protein array (RPPA) data from The Cancer Proteome Atlas (TCPA) was used to calculate the enrichment score. As to drug analysis, 265 aggregated small molecules were collected from Genomics of Drug Sensitivity in Cancer (GDSC, <https://www.cancerrxgene.org/>). Multi-omics data of pan-cancer was obtained from TCGA and UCSC Xena, and some corresponding explorations were conducted by GSCALite (http://bioinfo.life.hust.edu.cn/web/GSCALite/) web server (7). The present study honored the data access policies of each database.

**Expression and clinical features of necroptosis regulators**

70 necroptosis-related molecules were collected from previous studies (8, 9), which summarized relevant protein-coding genes comprehensively. Differentially expressed genes (DEGs) between normal (n=59) and tumor (n=535) tissues were selected using the R package “DESeq2” (10) with the thresholds of |log2(Fold Change)|>0.5 and an adjusted p<0.05, which were included in the following research. The correlations between expression and DNA promoter methylation (TSS200, TSS1500, ‘1stExone’ and 5’UTR) levels were firstly estimated. Then SNV frequency and types of the differentially expressed necroptosis regulators in pan-cancer were analyzed by cBioPortal (https://www.cbioportal.org/) and the R package “maftools” (11). The percentage of CNV was characterized by homozygous, heterozygous, amplification (gain) and deletion (loss). To validate the results estimated by the “DESeq2”, expression changes between 347 adjacent cases and 515 tumor cases from the Toil-processed data, and among different pathologic stages were analyzed. Hierarchical clustering was used to classify necroptosis-related molecules into multiple groups, determining potential functional categorizes and expression connections.

**Construction of necroptosis regulator phenotypes**

Based on the expression levels of necroptosis-related molecules, unsupervised average linkage K-means clustering analysis measured by Euclidean distance was utilized to identify novel necroptosis phenotypes in LUAD through the R package “ConsensusClusterPlus” (12). The model was repeated 200 times with 80% of resampling rate, random seed fixed. Differences of the clinical phenotypes including age, gender, smoking history, relapse situation and pathologic stage as well as necroptosis regulators expressions among the clustering subtypes were estimated.

**Correlation of TME immune cell abundance and subtypes**

A list of 91 immunomodulators such as chemokines, receptors, interleukins and interferons was obtained from existing studies (13, 14). Tumor immune dysfunction and exclusion (TIDE) scores (15) were calculated with our normalized corrected expression matrix through an online application (<http://tide.dfci.harvard.edu/>) (16). The TIDE prediction scores are associated with T-cell dysfunction in cytotoxic T lymphocytes (CTL)-high tumors and T-cell exclusion in CTL-low tumors. Higher TIDE scores indicate insensitive immune checkpoint inhibitor (ICI) responses. Tumor purity was inferred by the “Estimation of Stromal and Immune cells in Malignant Tumors using Expression data (ESTIMATE)” algorithm (17), which contains immune and stromal admixture. Gene sets of several immunocyte infiltrating levels predicted by TIMER, CIBERSORT, CIBERSORT-ABS, QUANTISEQ, MCP-counter and XCELL methods were retrieved from a published study (14) and Tumor Immune Estimation Resource (TIMER, https://cistrome.shinyapps.io/timer/) database (18). Signatures of immune pathways such as cytolytic activity, inflammation promoting and T-cell co-inhibition was collected from Luo et al (19). A seven-step cancer immunity cycle has been verified to profile the status of anti-cancer immunity including release of cancer cell antigens, cancer antigen presentation, priming and activation, trafficking of immune cells to tumors, infiltration of immune cells into tumors, recognition of cancer cells by T cells and killing of cancer cells (20). Gene information was acquired from Tracking Tumor Immunopenotype (TIP, <http://biocc.hrbmu.edu.cn/TIP/index.jsp>) (21). The overall levels in each step were quantified by single sample Gene Set Enrichment Analysis (ssGSEA) through the R package “GSVA” (22, 23). In addition, DNA methylation levels of tumor-infiltrating lymphocyte (MeTIL) based on markers cg20792833, cg20425130, cg23642747, cg12069309 and cg21554552 in the TCGA-LUAD cohort were evaluated individually in light of the protocols outlined in the literature (24), with normalization but free of centralization in PCA to calculate MeTIL scores.

**Epigenetic variation and regulon calling**

30 mutational signatures were evaluated by the R package “deconstructSigs” (25) at the cutoff of 6%. In details, silent mutations were excluded, and normalization of nucleic acid sequence performed by the “exome2genome” method. Somatic CNV profiles at the focal and arm levels were identified and localized by the GISTIC2.0 software through GenePattern (<https://www.genepattern.org/>) web server, with the thresholds of copy number amplifications/deletions of ±0.1; focal length cutoff of 0.5; confidence level of 99% and q-value less than 0.05. The job memory and wall time were set to 2GB and 4 hours respectively in one CPU machine. The values of CNV and mutation matrix being equal to 2 were considered as high balanced gain whereas those of -2 were considered as high balanced loss. Additionally, the changes of CNV among the necroptosis phenotypes at focal and arm-level were estimated respectively (26). We further applied the R package “RTN” (27) to construct transcriptional regulatory networks (regulons), referring to genes whose products induces or suppress target gene sets (28), including 8 overlapped master transcription factors (FOXM1, HOXA4, DACH1, EPAS1, ETV5, FOXA2, ATOH8 and SMAD6) identified in a previous research (29). This computation was conducted in a two-core environment with a 1000-permutation strategy to obviate correlations at a false discovery rate (FDR) cutoff of 0.00001. A bootsrapping approach excluded unconfident associations in 1000 times of resampling. Individual regulon activity was estimated by two-sided GSEA through the “tni.gsea2” function. Moreover, a panel of 15 candidate regulators (SIRT4, HDAC5, SIRT2, SIRT1, SIRT5, SIRT7, PHF8, HDAC1, HDAC10, HDAC8, HDAC6, EP300, SIRT6, EHMT2 and CLOCK) relevant to cancerous chromatin remodeling (30) was explored.

**Pathway enrichment quantification**

First, the correlations between necroptosis regulators and cancer-related pathways, which contained TSC/mTOR, RTK, RAS/MAPK, PI3K/AKT, Hormone ER, Hormone AR, EMT DNA Damage Response, Cell Cycle and Apoptosis, were investigated. Specifically, pathway scores are composed of the relative protein level of all positive regulatory components minus that of negative regulatory components (31). Samples were divided into low- and high-expression groups at the median cutoff. Active effects were exhibited when the score of high-expression group outstripped that of low-expression group (32). To reveal the regulatory status in a proteomic aspect, we quantified the semantic similarities of the differentially expressed necroptosis molecules using the R package “GOSemSim” (33). In the present study, similarities were measured by the geometric mean of biological process (BP) and molecular function (MF) from gene ontology (GO) terms with a graph-based strategy (34). Marker genes of another ten typical pathways including Cell Cycle, Hippo, MYC, NOTCH, NRF2, RAS, PI3K, TP53, Wnt and TGFβ were collected from Chen’s study (35), and enrichment scores were obtained by the ssGSEA algorithm. Gene Set Variation Analysis (GSVA) (23) based on the “c2.cp.kegg.v7.4.symbols.gmt” gene set downloaded from Molecular Signatures Database (MSigDB, <http://www.gsea-msigdb.org/gsea/index.jsp>) was then performed. Significantly activated or inhibited genes between each two clustering subtypes were detected by the R package “limma” (36) with the thresholds of FDR<0.01 and |log2(FC)|>0.3.

**Clinical significance of the NecroScore**

Patients were distinguished into low- and high-NecroScore groups at the optimal cutoff by the R package “survminer”. The correlations between the NecroScore and clinicopathologic variables were then examined. Decision curves were used to analyze the net benefits. A nomogram and calibration curves were employed to construct a combined prognostic diagnostic model. Along with tumor mutation burden (TMB), samples were classified into novel four subtypes: low-TMB & low-NecroScore, low-TMB & high-NecroScore, high-TMB & high-NecroScore, and high-TMB & low-NecroScore where the stratification of TMB was determined by the optimal cutoff as described above. The correlation between the NecroScore and TME was estimated with the data downloaded from TIMER database. Immunophenotypes including C1 (wound healing), C2 (IFN-γ dominant), C3 (inflammatory), C4 (lymphocyte depleted) and C6 (TGF-β dominant)were collected from Thorsson’s study (37). We further downloaded the immunophenoscore (IPS) of LUAD individuals in TCGA-LUAD cohort to predict anti-CTLA-4 and anti-PD-1 status from The Cancer Immunome Atlas (TCIA, <https://tcia.at/>) (13), and levels between scoring groups were then evaluated. Additionally, the efficiency of ICI therapy between the two scoring subtypes was analyzed.

**Mutation architecture between scoring groups**

Synonymous and non-synonymous mutation were defined by the “maf.silent” function of the R package “maftools” (11). Non-synonymous mutation counts of genes no less than 65 were included for difference test between low- and high-NecroScore groups. The built-in function “somaticInteractions” was applied to determine significant mutual exclusivity and co-occurrence.

**Drug discovery and mechanism of actions**

The correlation between necroptosis-related molecules and chemotherapeutic sensitivity of distinct drugs were analyzed in GSCALite as previously stated. The R package “pRRophetic” (38) was used to predict half maximal inhibitory concentration (IC50) of target chemotherapies for LUAD including cisplatin, docetaxel, erlotinib, gefitinib, lapatinib and paclitaxel, in the basis of ridge regression. LUAD cell lines with drug sensitivity were retrieved from GDSC. A nearest centroid classifier was developed based on the necroptosis phenotypes and the expression of relevant differentially expressed molecules, which was applied to distinguish cell lines into several subtypes (39). The area under the curve (AUC) differences of drug responses among these clusters were finally tested. Only compounds which were detected in more than 90% of the LUAD cell lines were included for analysis. K-NearestNeighbor (KNN) algorithm was employed to compute missing values. Furthermore, we identified differentially expressed genes (DEGs) between low- and high-NecroScore groups by the R package “limma” (36) with the thresholds of |log2(FC)|>1 and FDR<0.001. DEGs were subsequently input to CLUE (<https://clue.io/>) to identify underlying mechanism of actions (MoA) and drug targets. Candidate small molecules for LUAD treatment were collected from the study of Malta *et al* (40). In their study, 43 stemness-related compounds in TCGA LUAD cohort were reported, and 24 overlapped drugs and corresponding MoA were ultimately enrolled for oncoprint analysis.

**Statistical analysis**

All statistical analysis was performed by R (version 4.0.3). Mann-Whitney U test (also known as Wilcoxon rank-sum test) for non-normally distributed continuous data and unpaired t-test for normal distributed continuous data were used to estimate the difference between two groups. To compare three or more groups, Kruskal-Wallis (KW), one-way ANOVA and Welch one-way ANOVA were applied. As to categorical data, Fisher’s exact test was employed in the present study. Correlation investigations were measured by Pearson correlation coefficients. But some evidence from GSCALite was based on Spearman correlation coefficients. Log-rank test was used for generated Kaplan-Meier (KM) curves in survival analysis by the R packages “survminer” and “survival”. Permutation test was used to examine the correlation between the NecroScore and mutant frequency. Independent prognostic analysis was conducted by univariate and multivariate Cox proportional hazard regression, estimating hazard ratio (HR) and 95% confidence interval (CI) simultaneously. For all unadjusted comparisons, a two-tailed p<0.05 was considered having statistically significance.

**References**

1. Tomida S, Takeuchi T, Shimada Y, Arima C, Matsuo K, Mitsudomi T, et al. Relapse-Related Molecular Signature in Lung Adenocarcinomas Identifies Patients with Dismal Prognosis. *J Clin Oncol* (2009) 27(17):2793-9. Epub 2009/05/06. doi: 10.1200/JCO.2008.19.7053.

2. Wilkerson MD, Yin X, Walter V, Zhao N, Cabanski CR, Hayward MC, et al. Differential Pathogenesis of Lung Adenocarcinoma Subtypes Involving Sequence Mutations, Copy Number, Chromosomal Instability, and Methylation. *PLoS One* (2012) 7(5):e36530. Epub 2012/05/17. doi: 10.1371/journal.pone.0036530.

3. Staaf J, Jonsson G, Jonsson M, Karlsson A, Isaksson S, Salomonsson A, et al. Relation between Smoking History and Gene Expression Profiles in Lung Adenocarcinomas. *BMC Med Genomics* (2012) 5:22. Epub 2012/06/09. doi: 10.1186/1755-8794-5-22.

4. Okayama H, Kohno T, Ishii Y, Shimada Y, Shiraishi K, Iwakawa R, et al. Identification of Genes Upregulated in Alk-Positive and Egfr/Kras/Alk-Negative Lung Adenocarcinomas. *Cancer Res* (2012) 72(1):100-11. Epub 2011/11/15. doi: 10.1158/0008-5472.CAN-11-1403.

5. Yamauchi M, Yamaguchi R, Nakata A, Kohno T, Nagasaki M, Shimamura T, et al. Epidermal Growth Factor Receptor Tyrosine Kinase Defines Critical Prognostic Genes of Stage I Lung Adenocarcinoma. *PLoS One* (2012) 7(9):e43923. Epub 2012/10/03. doi: 10.1371/journal.pone.0043923.

6. Vivian J, Rao AA, Nothaft FA, Ketchum C, Armstrong J, Novak A, et al. Toil Enables Reproducible, Open Source, Big Biomedical Data Analyses. *Nat Biotechnol* (2017) 35(4):314-6. Epub 2017/04/12. doi: 10.1038/nbt.3772.

7. Liu CJ, Hu FF, Xia MX, Han L, Zhang Q, Guo AY. Gscalite: A Web Server for Gene Set Cancer Analysis. *Bioinformatics* (2018) 34(21):3771-2. Epub 2018/05/24. doi: 10.1093/bioinformatics/bty411.

8. Zhao Z, Liu H, Zhou X, Fang D, Ou X, Ye J, et al. Necroptosis-Related Lncrnas: Predicting Prognosis and the Distinction between the Cold and Hot Tumors in Gastric Cancer. *J Oncol* (2021) 2021:6718443. Epub 2021/11/19. doi: 10.1155/2021/6718443.

9. Park JE, Lee JH, Lee SY, Hong MJ, Choi JE, Park S, et al. Expression of Key Regulatory Genes in Necroptosis and Its Effect on the Prognosis in Non-Small Cell Lung Cancer. *J Cancer* (2020) 11(18):5503-10. Epub 2020/08/04. doi: 10.7150/jca.46172.

10. Love MI, Huber W, Anders S. Moderated Estimation of Fold Change and Dispersion for Rna-Seq Data with Deseq2. *Genome Biol* (2014) 15(12):550. Epub 2014/12/18. doi: 10.1186/s13059-014-0550-8.

11. Mayakonda A, Lin DC, Assenov Y, Plass C, Koeffler HP. Maftools: Efficient and Comprehensive Analysis of Somatic Variants in Cancer. *Genome Res* (2018) 28(11):1747-56. Epub 2018/10/21. doi: 10.1101/gr.239244.118.

12. Wilkerson MD, Hayes DN. Consensusclusterplus: A Class Discovery Tool with Confidence Assessments and Item Tracking. *Bioinformatics* (2010) 26(12):1572-3. Epub 2010/04/30. doi: 10.1093/bioinformatics/btq170.

13. Charoentong P, Finotello F, Angelova M, Mayer C, Efremova M, Rieder D, et al. Pan-Cancer Immunogenomic Analyses Reveal Genotype-Immunophenotype Relationships and Predictors of Response to Checkpoint Blockade. *Cell Rep* (2017) 18(1):248-62. Epub 2017/01/05. doi: 10.1016/j.celrep.2016.12.019.

14. Xiao Y, Ma D, Zhao S, Suo C, Shi J, Xue MZ, et al. Multi-Omics Profiling Reveals Distinct Microenvironment Characterization and Suggests Immune Escape Mechanisms of Triple-Negative Breast Cancer. *Clin Cancer Res* (2019) 25(16):5002-14. Epub 2019/03/07. doi: 10.1158/1078-0432.CCR-18-3524.

15. Jiang P, Gu S, Pan D, Fu J, Sahu A, Hu X, et al. Signatures of T Cell Dysfunction and Exclusion Predict Cancer Immunotherapy Response. *Nat Med* (2018) 24(10):1550-8. Epub 2018/08/22. doi: 10.1038/s41591-018-0136-1.

16. Fu J, Li K, Zhang W, Wan C, Zhang J, Jiang P, et al. Large-Scale Public Data Reuse to Model Immunotherapy Response and Resistance. *Genome Med* (2020) 12(1):21. Epub 2020/02/28. doi: 10.1186/s13073-020-0721-z.

17. Yoshihara K, Shahmoradgoli M, Martinez E, Vegesna R, Kim H, Torres-Garcia W, et al. Inferring Tumour Purity and Stromal and Immune Cell Admixture from Expression Data. *Nat Commun* (2013) 4:2612. Epub 2013/10/12. doi: 10.1038/ncomms3612.

18. Li T, Fan J, Wang B, Traugh N, Chen Q, Liu JS, et al. Timer: A Web Server for Comprehensive Analysis of Tumor-Infiltrating Immune Cells. *Cancer Res* (2017) 77(21):e108-e10. Epub 2017/11/03. doi: 10.1158/0008-5472.CAN-17-0307.

19. Luo Q, Vogeli TA. A Methylation-Based Reclassification of Bladder Cancer Based on Immune Cell Genes. *Cancers (Basel)* (2020) 12(10). Epub 2020/10/24. doi: 10.3390/cancers12103054.

20. Chen DS, Mellman I. Oncology Meets Immunology: The Cancer-Immunity Cycle. *Immunity* (2013) 39(1):1-10. Epub 2013/07/31. doi: 10.1016/j.immuni.2013.07.012.

21. Xu L, Deng C, Pang B, Zhang X, Liu W, Liao G, et al. Tip: A Web Server for Resolving Tumor Immunophenotype Profiling. *Cancer Res* (2018) 78(23):6575-80. Epub 2018/08/30. doi: 10.1158/0008-5472.CAN-18-0689.

22. Barbie DA, Tamayo P, Boehm JS, Kim SY, Moody SE, Dunn IF, et al. Systematic Rna Interference Reveals That Oncogenic Kras-Driven Cancers Require Tbk1. *Nature* (2009) 462(7269):108-12. Epub 2009/10/23. doi: 10.1038/nature08460.

23. Hanzelmann S, Castelo R, Guinney J. Gsva: Gene Set Variation Analysis for Microarray and Rna-Seq Data. *BMC Bioinformatics* (2013) 14:7. Epub 2013/01/18. doi: 10.1186/1471-2105-14-7.

24. Jeschke J, Bizet M, Desmedt C, Calonne E, Dedeurwaerder S, Garaud S, et al. DNA Methylation-Based Immune Response Signature Improves Patient Diagnosis in Multiple Cancers. *J Clin Invest* (2017) 127(8):3090-102. Epub 2017/07/18. doi: 10.1172/JCI91095.

25. Rosenthal R, McGranahan N, Herrero J, Taylor BS, Swanton C. Deconstructsigs: Delineating Mutational Processes in Single Tumors Distinguishes DNA Repair Deficiencies and Patterns of Carcinoma Evolution. *Genome Biol* (2016) 17:31. Epub 2016/02/24. doi: 10.1186/s13059-016-0893-4.

26. Shen R, Li P, Li B, Zhang B, Feng L, Cheng S. Identification of Distinct Immune Subtypes in Colorectal Cancer Based on the Stromal Compartment. *Front Oncol* (2019) 9:1497. Epub 2020/01/31. doi: 10.3389/fonc.2019.01497.

27. Fletcher MN, Castro MA, Wang X, de Santiago I, O'Reilly M, Chin SF, et al. Master Regulators of Fgfr2 Signalling and Breast Cancer Risk. *Nat Commun* (2013) 4:2464. Epub 2013/09/18. doi: 10.1038/ncomms3464.

28. Castro MA, de Santiago I, Campbell TM, Vaughn C, Hickey TE, Ross E, et al. Regulators of Genetic Risk of Breast Cancer Identified by Integrative Network Analysis. *Nat Genet* (2016) 48(1):12-21. Epub 2015/12/01. doi: 10.1038/ng.3458.

29. De Bastiani MA, Klamt F. Integrated Transcriptomics Reveals Master Regulators of Lung Adenocarcinoma and Novel Repositioning of Drug Candidates. *Cancer Med* (2019) 8(15):6717-29. Epub 2019/09/11. doi: 10.1002/cam4.2493.

30. Audia JE, Campbell RM. Histone Modifications and Cancer. *Cold Spring Harb Perspect Biol* (2016) 8(4):a019521. Epub 2016/04/03. doi: 10.1101/cshperspect.a019521.

31. Akbani R, Ng PK, Werner HM, Shahmoradgoli M, Zhang F, Ju Z, et al. A Pan-Cancer Proteomic Perspective on the Cancer Genome Atlas. *Nat Commun* (2014) 5:3887. Epub 2014/05/30. doi: 10.1038/ncomms4887.

32. Ye Y, Xiang Y, Ozguc FM, Kim Y, Liu CJ, Park PK, et al. The Genomic Landscape and Pharmacogenomic Interactions of Clock Genes in Cancer Chronotherapy. *Cell Syst* (2018) 6(3):314-28 e2. Epub 2018/03/12. doi: 10.1016/j.cels.2018.01.013.

33. Yu G, Li F, Qin Y, Bo X, Wu Y, Wang S. Gosemsim: An R Package for Measuring Semantic Similarity among Go Terms and Gene Products. *Bioinformatics* (2010) 26(7):976-8. Epub 2010/02/25. doi: 10.1093/bioinformatics/btq064.

34. Wang JZ, Du Z, Payattakool R, Yu PS, Chen CF. A New Method to Measure the Semantic Similarity of Go Terms. *Bioinformatics* (2007) 23(10):1274-81. Epub 2007/03/09. doi: 10.1093/bioinformatics/btm087.

35. Chen X, Chen H, Yao H, Zhao K, Zhang Y, He D, et al. Turning up the Heat on Non-Immunoreactive Tumors: Pyroptosis Influences the Tumor Immune Microenvironment in Bladder Cancer. *Oncogene* (2021) 40(45):6381-93. Epub 2021/10/01. doi: 10.1038/s41388-021-02024-9.

36. Ritchie ME, Phipson B, Wu D, Hu Y, Law CW, Shi W, et al. Limma Powers Differential Expression Analyses for Rna-Sequencing and Microarray Studies. *Nucleic Acids Res* (2015) 43(7):e47. Epub 2015/01/22. doi: 10.1093/nar/gkv007.

37. Thorsson V, Gibbs DL, Brown SD, Wolf D, Bortone DS, Ou Yang TH, et al. The Immune Landscape of Cancer. *Immunity* (2018) 48(4):812-30 e14. Epub 2018/04/10. doi: 10.1016/j.immuni.2018.03.023.

38. Geeleher P, Cox N, Huang RS. Prrophetic: An R Package for Prediction of Clinical Chemotherapeutic Response from Tumor Gene Expression Levels. *PLoS One* (2014) 9(9):e107468. Epub 2014/09/18. doi: 10.1371/journal.pone.0107468.

39. Ho KH, Huang TW, Liu AJ, Shih CM, Chen KC. Cancer Essential Genes Stratified Lung Adenocarcinoma Patients with Distinct Survival Outcomes and Identified a Subgroup from the Terminal Respiratory Unit Type with Different Proliferative Signatures in Multiple Cohorts. *Cancers (Basel)* (2021) 13(9). Epub 2021/05/01. doi: 10.3390/cancers13092128.

40. Malta TM, Sokolov A, Gentles AJ, Burzykowski T, Poisson L, Weinstein JN, et al. Machine Learning Identifies Stemness Features Associated with Oncogenic Dedifferentiation. *Cell* (2018) 173(2):338-54 e15. Epub 2018/04/07. doi: 10.1016/j.cell.2018.03.034.
